# Validity and Reliability of the Turkish Version of the Idiopathic Toe Walking Outcome (iTWO) Proforma

**DOI:** 10.1002/jfa2.70188

**Published:** 2026-07-28

**Authors:** Esra Giray, Munteha Ilkyaz Karadag, Sude Gozukucuk Turkyilmaz, Kubra Yildiz Celik, Pinar Akpinar, Ozge Gulsum Illeez, Feyza Unlu Ozkan, Ilknur Aktas, Evrim Karadag‐Saygi, Cylie M. Williams

**Affiliations:** ^1^ Department of Physical Medicine and Rehabilitation University of Health Sciences Fatih Sultan Mehmet Training and Research Hospital Istanbul Türkiye; ^2^ Department of Physical Medicine and Rehabilitation University of Health Sciences Sisli Hamidiye Etfal Training and Research Hospital Istanbul Türkiye; ^3^ Department of Physical Medicine and Rehabilitation Marmara University School of Medicine Istanbul Türkiye; ^4^ School of Primary and Allied Health Care Monash University, Peninsula Campus Frankston Victoria Australia

**Keywords:** 6‐minute walk test, idiopathic toe walking, Toe Walking Severity Scale, treatment outcomes, weight‐bearing lunge

## Abstract

**Introduction:**

This study aims to evaluate the validity and reliability of the Turkish version of the Idiopathic Toe Walking Outcome proforma (iTWO proforma‐T), a clinician‐ and parent‐reported tool designed to assess symptoms, functional performance, and treatment outcomes in children with idiopathic toe walking (ITW).

**Methods:**

Thirty‐five children aged 4–14 years with ITW were included. The translation procedure followed the International Society for Pharmacoeconomics and Outcomes Research (ISPOR) guidelines. Construct validity was examined through correlations between individual iTWO proforma‐T items and the Toe Walking Severity Scale, the Oxford Ankle Foot Questionnaire (OxAFQ), and the Pediatric Outcomes Data Collection Instrument (PODCI). Test–retest reliability was assessed in 10 of the 35 participants using intraclass correlation coefficients (ICC).

**Results:**

Most items demonstrated excellent test–retest reliability (ICC > 0.90), including parent‐reported toe‐walking percentage, satisfaction, treatment adherence, pain‐on‐activity, and clinician‐reported gait scale. Validity analyses showed significant and meaningful correlations between iTWO proforma‐T items and established measures. The estimated toe‐walking percentage correlated strongly with the Toe Walking Severity Scale (*ρ* = −0.549) and the OxAFQ emotional domain (*ρ* = −0.493). Functional performance items (gait scale, 2‐ and 6‐minute walk tests) showed moderate positive correlations with toe‐walking severity, whereas pain‐related items correlated negatively with relevant PODCI and OxAFQ domains.

**Conclusion:**

The iTWO proforma‐T is a valid and reliable instrument for evaluating symptoms, functional status, and treatment‐related outcomes in children with idiopathic toe walking. The tool preserves the structure and measurement characteristics of the original proforma and can be confidently used by clinicians and researchers for evidence‐based assessment and follow‐up in Turkish‐speaking pediatric populations.

## 1 Introduction

Toe walking is a gait pattern characterized by predominant forefoot loading during initial contact, resulting in absent or reduced heel strike [1]. Ongoing toe walking can lead to gastrocnemius contracture and lower limb pain [2]. The ongoing gait demands can variably impact children's participation in physical activity and their quality of life [3, 4]. This means that when treating children with toe walking, clinicians should use tools that capture important information. This is challenging with children who toe walk as it is caused by many reasons, and there are few assessment and outcome tools specific to this presentation.

The initial assessment of a child with toe walking should include a careful history and physical examination, focusing on development, onset, and progression of toe walking [5]. Idiopathic toe walking (ITW), as an exclusionary diagnosis, requires careful assessment to rule out neurological, neuromuscular, developmental, or primary orthopedic disorders associated with toe walking [6, 7]. There is ongoing debate regarding the relationship between autism spectrum disorder and ITW; however, most researchers in the field agree that these should be considered separate clinical entities [8]. ITW is often observed within families, indicating that genetic factors may contribute to its occurrence [9]. The prevalence of ITW is estimated to be around 5%, and most cases reportedly show a spontaneous reduction in toe walking over the years [10]. However, persistent ITW continues to be a concern for children and their families.

There are various treatment strategies suggested for ITW, including stretching exercises, motor control interventions, orthopedic footwear, serial casting, orthotic devices, Botulinum toxin type A injections, and soft‐tissue surgical procedures [11, 12]. These interventions primarily aim to preserve or improve ankle dorsiflexion and optimize gait function [12]. Because these outcomes are most measured in research, clinicians often primarily use the same measures as treatment outcomes. Focusing solely on these measures may overlook other factors that influence the child's and family's daily life, potentially hindering comprehensive follow‐up of this patient population and making treatment decisions more challenging.

There is emerging consensus working in some world regions about the treatment for ITW [13]. Yet, there is no standardized method of assessing outcomes recommended. This lack of consistency makes it difficult to compare results and evaluate the effectiveness of different interventions in this field. Recently, a consensus‐based core set of reliable outcome measures for assessing and monitoring treatment in children with ITW was developed [14]. This set of core measures was also validated through parents' perspectives on this core set, leading to the development of the ITW Treatment Outcomes proforma (iTWO proforma). However, validity and reliability of iTWO proforma have not been studied. Moreover, no comprehensive Turkish assessment tool is currently available for evaluating children with idiopathic toe walking. Therefore, this study aimed to translate the iTWO proforma into Turkish (iTWO proforma‐T‐Turkish version) and establish its validity and reliability.

## 2 Materials and Methods

This study is a correlational validation study of the Turkish language proforma for ITW. Ethical approval was obtained from the University of Health Sciences (Approval No: 10/40, Date: 08.05.2025). Informed consent was obtained from all legal guardians of the participating children and children assented to participation.

### 2.1 Participants

Children aged between 4 and 14 years with ITW and persistent toe‐walking gait were included. The ITW exclusionary diagnosis was confirmed by clinical evaluation by both a pediatric neurologist and physiatrist, supported by normal brain and spinal MRI. Also, when the physical examination suggested possible alternative causes, additional investigations, such as electroneuromyography and genetic and metabolic testing, were performed to exclude neurological, genetic, or metabolic disorders. These examinations ensured that participating children had no neurological, orthopedic, or developmental conditions associated with toe walking. Children were allowed to participate if they had prior treatment for ITW (including exercises, serial casting, orthosis, or botulinum toxin injection). Children were excluded from participation if they were still undergoing testing to determine any reasons for their toe walking.

### 2.2 Translation Procedure

The iTWO proforma‐T development team gave permission for the Turkish version study. The translation procedures were conducted in accordance with the ISPOR (International Society for Pharmacoeconomics and Outcomes Research) guidelines, following the steps of forward translation, back translation, harmonization, pilot testing, review, revision, and preparation of the final version of the questionnaire [15]. Accordingly, the iTWO proforma‐T, originally in English, was translated into Turkish by two independent researchers. Then, a bilingual expert fluent in both languages translated it back into English, and the versions were compared. After completing the back‐translation stage, the translators were brought together to discuss differences in terminology. The prefinal version was then pilot tested with 10 families to ensure that all items were clearly understood as intended. Based on the feedback obtained, minor revisions were made, and the final Turkish version of the questionnaire was established in order for it to be tested with clinicians and families.

### 2.3 Procedure and Outcomes

Recruitment occurred between June 2025 and November 2025 at the pediatric rehabilitation unit of the Physical Medicine and Rehabilitation Clinic, University of Health Sciences Fatih Sultan Mehmet Research and Training Hospital. Once the exclusionary ITW diagnosis was confirmed and consent obtained, demographic and clinical characteristics of all participants, including age, gender, body mass index, gestational week, type of delivery, age of independent walking, age at the beginning of toe walking, presence of family history for ITW, and Toe Walking Severity Scale scores, were recorded by the clinician. In all children, passive ankle dorsiflexion range of motion was measured in the supine position with the knee in extension using a standard clinical method [16], and ankle dorsiflexion was also assessed during the weight‐bearing lunge test using a tiltmeter application, as specified in the ITW proforma and previously described by Williams et al. [14, 17]. Toe Walking Severity Scale is a tool used to assess toe walking severity based on parental reports of the proportion of the child's daily ambulation spent walking on the toes. The scale comprises six grades: Grade 1 indicates toe walking during 76%–100% of ambulation; Grade 2, 51%–75%; Grade 3, 26%–50%; Grade 4, 10%–25%; Grade 5 reflects predominant plantar weight‐bearing with early heel rise, corresponding to occasional toe walking (< 10%); and Grade 6 represents a normal gait pattern with initial heel contact. The scale is adapted from the International Classification of Functioning, Disability, and Health—Children and Youth (ICF‐CY; WHO, 2001) and has been previously used to assess toe walking severity in children with idiopathic toe walking [11, 18, 19].

iTWO proforma‐T contains a set of outcome measures developed by healthcare professionals to be used during ITW treatment [14]. The Turkish version of the questionnaire was administered face‐to‐face to children with idiopathic toe walking who consented to participate in the study and the Turkish version is provided as Supporting Information S1. To assess the test–retest reliability, the questionnaire was re‐administered to 10 participants 1–3 days after the initial application. In the questionnaire, the first three items were directed to the parent or caregiver. The first item asked the parent to indicate the percentage of time their child spent toe walking over a self‐determined observation period. The second item asked the parent to rate their satisfaction with the child's toe walking on a 10‐point scale. The third item requested the parent to indicate the percentage of adherence to the prescribed treatment. The child was asked two questions regarding pain intensity, one concerning the current day and one during activity. The child selected a face on a visual analog scale, ranging from 0 to 10, to represent their pain level. The clinician performed ankle range of motion measurements using the weight‐bearing lunge test at both ankles and conducted a visual gait analysis according to the instructions specified in the iTWO proforma‐T gait scale item (Supporting Information S1) [14, 17]. Ankle dorsiflexion during the weight‐bearing lunge test was measured using a digital inclinometer or the TiltMeter application placed on the posterior aspect of the Achilles tendon, which has been shown to have high intra and inter‐rater reliability as previously described by Williams et al. [17]. In the gait analysis, the child was asked to attempt 10 heel‐walking steps while maintaining posture, and the appropriate stage indicating the severity of toe walking was graded on a 0–4 scale (0 = unable to touch heel/midfoot to floor; 4 = heel‐walking with forefoot lifted for 10 steps) as per the proforma's instructions. Subsequently, a 6‐minute walk test (6MWT) was performed, and distances were recorded in meters at the second, fourth, and sixth minutes.

The Oxford Ankle Foot Questionnaire (OxAFQ) and the Pediatric Outcomes Data Collection Instrument (PODCI) were administered to evaluate the validity of the iTWO proforma‐T. OxAFQ consists of items specific to the ankle and foot region and reflects the child's perspective on their health status. We used the Turkish validation of the OxAFQ [20]. Both parent and child‐reported versions of the questionnaire were administered. It includes a total of 15 questions covering the domains of physical function (OxAFQ‐PF), school and play (OxAFQ‐SP), emotional well‐being (OxAFQ‐EWB), and footwear (OxAFQ‐FW). Scores obtained for each domain were converted into a percentage format for analysis, with the higher number indicating a higher quality of life. For each child, eight subscale scores were recorded, derived from the four domains of both the child‐ and parent‐reported versions of the questionnaire.

The Pediatric Outcomes Data Collection Instrument, which is a parent‐reported outcome measure, was specifically developed to evaluate changes following pediatric orthopedic interventions across a broad range of diagnoses. Its items focus on aspects of the child's function and quality of life that may be influenced by treatment. The validity and reliability of the Turkish version of the questionnaire were previously established [21]. PODCI has six subscales: sports and physical function (PODCI‐SPF), pain/comfort (PODCI‐PC), upper extremity function (PODCI‐UE), transfer and basic mobility (PODCI‐TBM), happiness (PODCI‐H), and global functioning scale (PODCI‐GF). Normative and standardized scores vary between 0 and 100. “0” rates the worst and “100” rates the best status for each subscale.

### 2.4 Statistical Analysis

In accordance with the formula for correlational studies, considering a significance level of 0.05, a power of 0.80, and an anticipated correlation coefficient of 0.50 between scores on the 7‐item proforma and validated measures, a minimum of 20 participants should be included in the study. Given that the Turkish iTWO proforma‐T consists of 7 items, various statistical methods guided the determination of sample size. For assessing internal consistency using Cronbach's alpha, it is recommended to have at least 5–10 participants per item, suggesting a minimum of 35–70 participants [22].

IBM SPSS Statistics Version 26 for Mac was used to analyze data. Data distribution was assessed with histograms, normality plots, and the Shapiro–Wilk normality test. Descriptive analyses were used to present demographic characteristics and the basic features of the data. Although both left and right ankle range of motion were collected to ensure minimal differences, only the right limb measures were presented in correlation analyses. As ITW is known as a symmetrical condition, using the right only satisfies independence due to the likelihood of high correlations between limbs [23].

To evaluate test–retest reliability of iTWO proforma‐T, the intraclass correlation coefficient (ICC, range 0.00–1.00) was calculated for each item. ICC values less than 0.5 were considered as poor reliability, values between 0.5 and 0.75 were considered as moderate reliability, values between 0.75 and 0.9 were considered as good reliability, and values greater than 0.90 were considered as excellent reliability [24, 25]. To investigate the validity of the iTWO proforma‐T, Spearman correlation analysis was used to assess the correlation between individual iTWO proforma‐T items and the Toe Walking Severity Scale, OxAFQ, and the PODCI subitems. Spearman correlation coefficients (*ρ*) were classified as follows: < 0.30 = small/negligible; 0.30–0.50 = low; 0.50–0.70 = moderate; 0.70–0.90 = high; and > 0.90 = very high [25, 26]. A *p*‐value of < 0.05 was considered statistically significant.

## 3 Results

**FIGURE 1:**
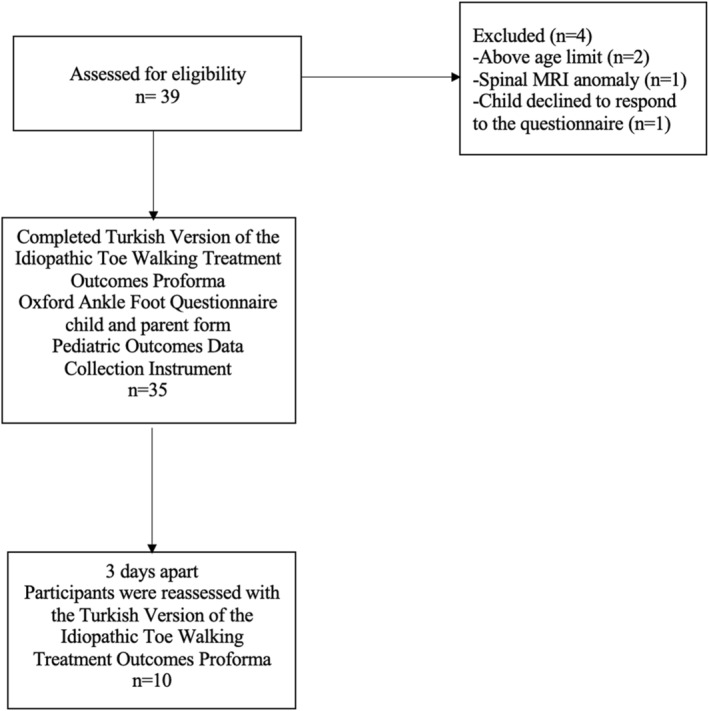
The CONSORT flow diagram showing participant recruitment.

Of the 39 children assessed for eligibility, 35 met the inclusion criteria and consented to participate in this study. The CONSORT flow diagram illustrating participant recruitment is shown in Figure 1. Demographic characteristics of the participants are presented in Table 1.

**TABLE 1:** Demographic characteristics of 35 children.

	Mean (SD), number (%)
Age (years)	9.2 (2.59)
Gender	
Female	14 (40%)
BMI (kg/m^2^)	20.4 (3.67)
Type of delivery	
Cesarean	18 (51%)
Vaginal	17 (49%)
Gestational weeks	38.1 (2.96)
Age of ındependent walking	12.9 (2.24)
Age at the beginning of toe walking (month)	33.7 (22.5)
Presence of family history for ITW	7 (20%)
Grade of toe walking severity	
Grade 1	9 (25.7%)
Grade 2	10 (28.6%)
Grade 3	8 (22.9%)
Grade 4	5 (14.3%)
Grade 5	3 (8.6%)
Grade 6	0
Weight bearing lunge measure of ankle dorsiflexion (°)	
Right—knee straight	21.37 (6.14)
Right—knee bent	29.37 (7.73)
Left—knee straight	19.29 (6.71)
Left—knee bent	27.46 (9.19)
Ankle passive dorsiflexion range of motion	
Right	11.5 (6.85)
Left	11.7 (7.06)

### 3.1 Validity and Reliability Assessment

Table 2 presents the results for the domains of iTWO proforma‐T, including the descriptive measures for each domain. Most items showed excellent test–retest reliability (Table 3). Question 1–3, Question 4 (activity), weight‐bearing lunge knee straight, and the Gait scale items all had ICC values above 0.90 (Table 3). Since the iTWO proforma‐T does not provide a total score, validity analyses were conducted for each individual item (with right leg measures only) rather than for an overall composite score.

**TABLE 2:** Descriptive statistics for the items of the Idiopathic Toe Walking Outcome proforma, the Oxford Ankle Foot Questionnaire (OxAFQ), and the Pediatric Outcomes Data Collection Instrument (PODCI).

	Min	Max	Median
Question 1: Estimate how much time has your child spent toe walking in the last…Day or week?	1	10	5
Question 2: How satisfied are you with this amount of toe walking?	0	10	0
Question 3: How well you have been able to follow the treatment since last visit?	0	10	4
Question 4 (today): Ask the child “What is the worst pain you get in your legs today?”	0	8	2
Question 4 (activity): Ask the child “What is the worst pain you get when moving about in the last week?”	0	10	2
WBL, knee straight right	5	31	22.5
WBL, knee straight left	6	35	20.0
WBL, knee bent right	13	49	29.5
Gait scale	2	4	4
2 minute	15	204	126.5
4 minute	20	252	134
6 minute	13	180	132
PODCI global function score	66	100	95
PODCI upper extremity and physical functioning	20	100	96
PODCI transfer and basic mobility	85	100	100
PODCI sports and physical functioning	53	100	93
PODCI pain/comfort	33	100	89
PODCI happiness	50	100	100
OxFAQ physical function (child)	25	100	79.1
OxFAQ school and play (child)	31.2	100	100
OxFAQ emotional well‐being (child)	25	100	81.2
OxFAQ footwear (child)	25	100	100
OxFAQ physical function (parent)	25	100	79.1
OxFAQ school and play (parent)	50	100	100
OxFAQ emotional well‐being (parent)	6	100	93.7
OxFAQ footwear (parent)	25	100	100

Abbreviations: OxFAQ, Oxford Foot Ankle Questionnaire; PODCI, Pediatric Outcomes Data Collection Instrument.

**TABLE 3:** Test–retest reliability of iTWO proforma‐T items.

	ICC	Lower bound	Upper bound
Question 1: Estimate how much time has your child spent toe walking in the last…Day or week?	0.993	0.971	0.998
Question 2: How satisfied are you with this amount of toe walking?	0.965	0.866	0.991
Question 3: How well you have been able to follow the treatment since last visit?	0.934	0.758	0.983
Question 4 (today): Ask the child “What is the worst pain you get in your legs today?”	0.672	0.117	0.907
Question 4 (activity): Ask the child “What is the worst pain you get when moving about in the last week?”	0.951	0.816	0.988
WBL, knee straight right	0.953	0.824	0.988
WBL, knee straight, left	0.828	0.452	0.954
WBL, knee bent, right	0.687	0.144	0.912
WBL, knee bent, left	0.719	0.207	0.922
Gait scale	1.000	1.000	1.000
2 minute	0.789	0.355	0.943
4 minute	0.683	0.138	0.911
6 minute	0.864	0.546	0.964

Abbreviations: ICC, intraclass correlation coefficient; WBL, weight bearing lunge.

**FIGURE 2:**
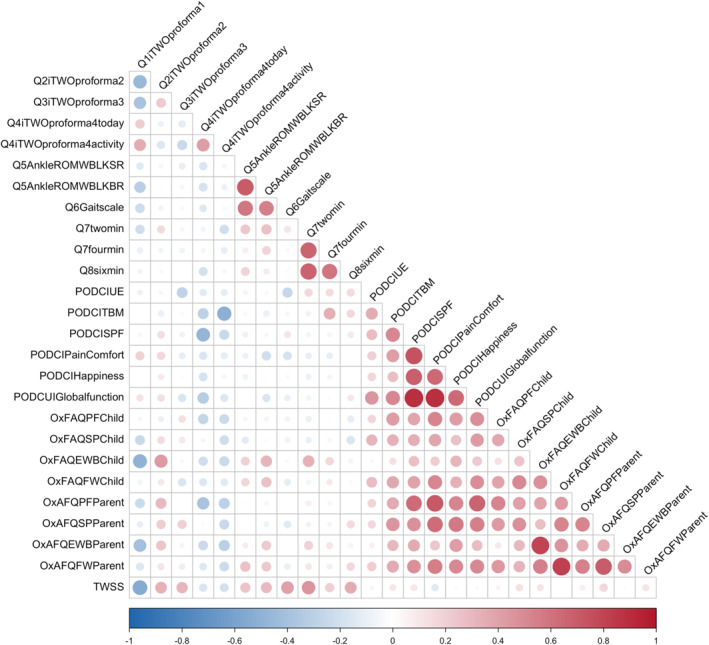
Heat map of Spearman correlation coefficients between individual ITW proforma items and established outcome measures (PODCI, Oxford Ankle Foot Questionnaire, and Toe Walking Severity Scale). Only the lower triangular portion of the matrix is shown to avoid redundancy. Circle size and color intensity represent the strength of correlations, with red indicating negative and blue indicating positive associations. The full correlation matrix with numerical values is presented in Table S1.

Figure 2 and Table S1 present the correlations between the individual iTWO proforma‐T items and the Toe Walking Severity Scale, OxAFQ child and parent scores, and PODCI subitems, demonstrating the construct validity of the instrument. There were a number if iTWO proforma‐T items that had moderate correlations including, the amount of toe walking which negatively correlated with parent satisfaction with amount of toe walking (*ρ* = −0.457, *p* = 0.006), and showed a low‐to‐moderate negative correlation with ability to adhere to treatment (*ρ* = −0.382, *p* = 0.024).

The Gait Scale, 2‐minute, and 6‐minute walk tests demonstrated low positive correlations with the Toe Walking Severity Scale (*ρ* = 0.403, 0.459, and 0.353, respectively), indicating that poorer performance on these functional measures is associated with greater toe‐walking severity. The Q4A item (pain on activity in the legs) showed a negative moderate correlation with the PODCI Transfer and Basic Mobility domain (*ρ* = −0.514), suggesting that children who report greater difficulty in daily activities on the ITWO also tend to have more limitations in mobility and transfers. Similarly, the Q4T item (pain today in the legs) correlated negatively with both the PODCI Physical Functioning and Sports domain (*ρ* = −0.470) and the parent‐reported OxAFQ Physical domain (*ρ* = −0.381), reinforcing that the physical challenges identified by the iTWO proforma‐T are consistent with both clinician‐rated and parent‐observed functional limitations. Interitem correlation analysis revealed moderate‐to‐high correlations among weight‐bearing lunge measurements with the knee straight (right and left), weight‐bearing lunge measurements with the knee bent (right and left), and the gait scale, reflecting shared measurement characteristics.

## 4 Discussion

The present study demonstrated that the iTWO proforma‐T is a valid and reliable instrument for evaluating treatment outcomes of children with ITW. The correlations demonstrate that further work is needed on this proforma to determine whether all measures are necessary or only those items with the most consistent responses and that are meaningful to parents should be retained. The current study demonstrates that this structure can be applied effectively across diverse cultural and linguistic settings [14]. Although Gray et al.'s study did not include formal psychometric validation, the present study extends the evidence base by providing detailed reliability and validity metrics, thereby contributing novel data to the literature and informing future research directions.

Heterogeneous outcome measures and variable follow‐up periods make it difficult to compare treatment effectiveness across studies on ITW, and the overall methodological quality of the available research remains limited. These limitations underscore the need for standardized and clinically meaningful assessment tools. In this context, the iTWO proforma‐T provides a structured way to evaluate toe walking symptoms, functional performance, and treatment‐related experiences, helping to address some of the gaps noted in earlier work.

Most items of the instrument showed excellent test–retest reliability in our study, whereas the remaining domains demonstrated good‐to‐moderate stability over time. These findings suggest that the parent‐reported domains, such as estimated toe‐walking time, parental satisfaction, and treatment adherence, are stable constructs that can be reliably assessed in routine clinical practice. Similarly, clinician‐reported items, such as the gait severity scale and weight‐bearing lunge test (knee straight), also exhibited excellent reliability, reinforcing their suitability for monitoring changes over time. Moderate reliability observed in the knee‐bent lunge test may be attributable to greater postural instability during this position compared with the knee‐straight position. Similarly, the moderate reliability observed for the 4‐minute walk velocity may reflect the performance‐based nature of this measure, which depends on the child's motivation, attention, and pacing strategy. Evidence regarding a learning effect on walk‐test performance in children remains mixed; however, some studies have reported that encouragement and repeated exposure to the test can influence walking distance and pacing behavior, which may partly explain the variability observed between test and retest sessions in the present study [27]. Construct validity of the iTWO proforma‐T was supported through significant and meaningful correlations with established outcome measures, including the Toe Walking Severity Scale, OxAFQ, and PODCI. Finding correlations between such items as amount of toe walking, satisfaction with treatment and adherence warrant further exploration. Functional performance item correlations highlight the challenges and variability in data collection with young children and should be viewed cautiously.

A strength of this study is that it represents the first validation and reliability study of the iTWO proforma as the original English version had not previously undergone formal psychometric evaluation. Therefore, this study provides the first evidence of the instrument's validity and reliability, while simultaneously introducing its first Turkish translation, thereby making a culturally adapted and psychometrically evaluated tool available to Turkish‐speaking clinicians and families. The translation process itself was rigorous and helped ensure that the questionnaire was easy to understand for both clinicians and parents. A limitation of this study is that the proforma did not provide a total score; if a total score were available, it would make the tool more convenient for use in clinical practice or in studies comparing treatment effects or monitoring changes over time. Another limitation is that some correlations between the quality‐of‐life measures and the iTWO proforma‐T items were low or absent, which may be attributable to differences in item content or construct overlap between the instruments.

Future studies should focus on developing a total score for the iTWO proforma‐T and establishing the minimal clinically important difference (MCID) for both the total score and individual items, in order to facilitate its use in evaluating treatment effectiveness and monitoring clinically meaningful change over time.

## 5 Conclusion

In conclusion, the Turkish version of the iTWO proforma‐T was found to be a valid and reliable tool for assessing toe walking‐related symptoms, functional performance, and treatment‐related experiences in children with idiopathic toe walking. This study represents the first psychometric evaluation of the iTWO proforma, providing evidence for its validity and reliability, and offers clinicians and researchers a culturally adapted, Turkish‐language instrument for evidence‐based evaluation, clinical follow‐up, and outcome monitoring in children with ITW.

## Author Contributions

**Esra Giray:** conceptualization, data curation, formal analysis, investigation, methodology, project administration, resources, software, supervision, validation, visualization, writing – original draft, writing – review and editing. **Munteha Ilkyaz Karadag:** conceptualization, data curation, formal analysis, investigation, methodology, project administration, resources, software, supervision, validation, visualization, writing – original draft, writing – review and editing. **Sude Gozukucuk Turkyilmaz:** conceptualization, data curation, formal analysis, investigation, methodology, project administration, resources, software, supervision, validation, visualization, writing – original draft, writing – review and editing. **Kubra Yildiz Celik:** conceptualization, data curation, formal analysis, investigation, methodology, project administration, resources, software, supervision, validation, visualization, writing – original draft, writing – review and editing. **Pinar Akpinar:** conceptualization, data curation, formal analysis, investigation, methodology, project administration, resources, software, supervision, validation, visualization, writing – original draft, writing – review and editing. **Ozge Gulsum Illeez:** conceptualization, data curation, formal analysis, investigation, methodology, project administration, resources, software, supervision, validation, visualization, writing – original draft, writing – review and editing. **Feyza Unlu Ozkan:** conceptualization, data curation, formal analysis, investigation, methodology, project administration, resources, software, supervision, validation, visualization, writing – original draft, writing – review and editing. **Ilknur Aktas:** conceptualization, data curation, formal analysis, investigation, methodology, project administration, resources, software, supervision, validation, visualization, writing – original draft, writing – review and editing. **Evrim Karadag‐Saygi:** conceptualization, data curation, formal analysis, investigation, methodology, project administration, resources, software, supervision, validation, visualization, writing – original draft, writing – review and editing. **Cylie M. Wıllıams:** conceptualization, data curation, formal analysis, investigation, methodology, project administration, resources, software, supervision, validation, visualization, writing – original draft, writing – review and editing. All authors have read and agreed to the published version of the manuscript.

## Funding

The authors have nothing to report.

## Ethics Statement

Ethical approval was obtained from Scientific Ethics Committee of University of Health Sciences (Approval No: 10/40, Date: 08.05.2025).

## Consent

Informed consent was obtained from all legal guardians of the participating children and children assented to participation.

## Conflicts of Interest

The authors declare no conflicts of interest.

## Permission to Reproduce Material From Other Sources

All figures and tables are original and were created by the authors. No copyrighted material from other sources was reproduced or adapted.

## Supporting information


Supporting Information S1

**Table S1:** Correlation analyses demonstrating the validity of the iTWO proforma‐T.

## Data Availability

The data that support the findings of this study are available on request from the corresponding author (EG). The data are not publicly available due to their containing information that could compromise the privacy of research participants.
