# Supplementary material for: Validity and Reliability of the Turkish Version of the Idiopathic Toe Walking Outcome (iTWO) Proforma
Source: J Foot Ankle Res. 2026 Jul 28;19(3):e70188. doi: 10.1002/jfa2.70188 (PMC13415977; doi:10.1002/jfa2.70188)
Supplement: Supplementary file 1 — Supporting Information S1 [file JFA2-19-e70188-s001.docx]

İdiyopatik Parmak Ucu Yürüyüşü Tedavi Sonuçları Formu (IPUY-TSF)

**Ebeveyn tarafından bildirilen ölçümler – (Ebeveyn / bakım veren aşağıdaki soruları uygun şekilde dolduracaktır)**

1. Çocuğunuzun son... gün veya hafta boyunca ne kadar süre parmak ucunda yürüdüğünü tahmin edin. (Lütfen gün veya haftayı daire içine alın ve çizgi üzerine bir X işareti koyun)

*___________________________________________________*

            %0      %10       %20      %30       %40       %50     %60       %70       %80       %90     %100

     2. Bu parmak ucunda yürüme sıklığından ne kadar memnunsunuz? (Lütfen çizgi üzerine bir X işareti koyun)

            __________________________________________________

               0           1              2             3             4            5           6            7            8           9           10

             Hiç Memnun Değilim                            Memnun veya Memnuniyetsiz Değilim                   Tamamen Memnunum

  3. Son ziyaretten bu yana tedaviyi ne kadar iyi uygulayabildiniz? (Lütfen çizgi üzerine işaretleyin ve eğer birden fazla tedavi varsa, bir X işareti koyun ve hangi tedavi olduğunu yazın ve tüm tedaviler için tekrarlayın)

*___________________________________________________*

0%      10%       20%       30%        40%       50%      60%       70%       80%       90%      100%

                     Hiç uygulayamadı                        Zamanın yarısında uygulayabildi                    Her zaman uygulayabildi

4.  **Bugünkü** ağrı: Uygun ölçeğe **B** işareti koyun: Çocuğun yanıtı

     Çocuğa ‘Bugün bacaklarında hissettiğin en kötü ağrı nedir?’ diye sorun.

**Aktivite** sırasında ağrı: Uygun ölçeğe **A** işareti koyun: Çocuğun yanıtı

     Çocuğa ‘ Son bir haftada hareket halindeyken hissettiğin en kötü ağrı nedir? ‘ diye sorun.


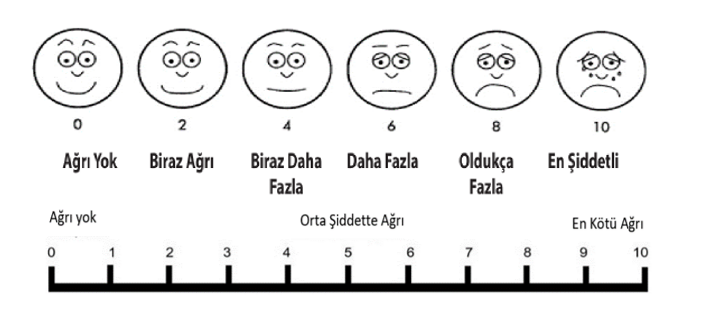


*Klinisyene not:*

*Çocuğu yaşına / kapasitesine göre bir yüzü veya VAS’ı seçin*

***Değerlendirmeler***

***Ayak Bileği Eklem Hareket Açıklığı***

Ağırlık taşımayan yöntemle (ATL) (dijital inklinometre veya ölçüm uygulaması kullanarak) ya da klinik olarak uygun ise ağırlık aktarımlı lunge (hamle) (AAL) sırasında ölçün.


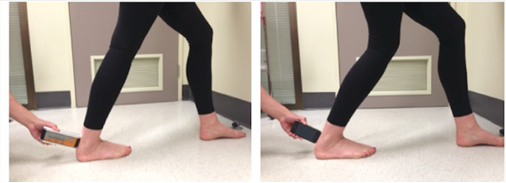


Journal of Science and Medicine in Sport, Cilt 16, Sayı 5, Williams C.M ve ark., "The TiltMeter app is a novel and accurate measurement tool for the weight bearing lunge test" başlıklı makaleden yeniden basılmıştır, Sayfa 392-395, Telif Hakkı (2013), Elsevier'den izin alınarak.

| Metodu daire içine alın: | **Sağ** | **Sol** | Metodu Daire İçine Alın | **Sağ** | **Sol** |
| --- | --- | --- | --- | --- | --- |
| AAL /ATL- dizler düz |  |  | AAL/ATL- dizler bükülü |  |  |

***Yürüme Ölçeği*** *(Gerekirse klinisyen uygulamalı olarak gösterebilir)*

Çocuğa "Ayakların arasında normal bir mesafe olacak şekilde ve vücut pozisyonunu değiştirmemeye çalışarak, topuklarının üzerinde 10 adım atabilir misin?" diye sorun.

| 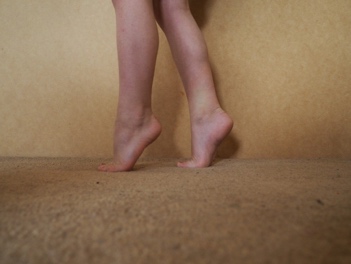 | 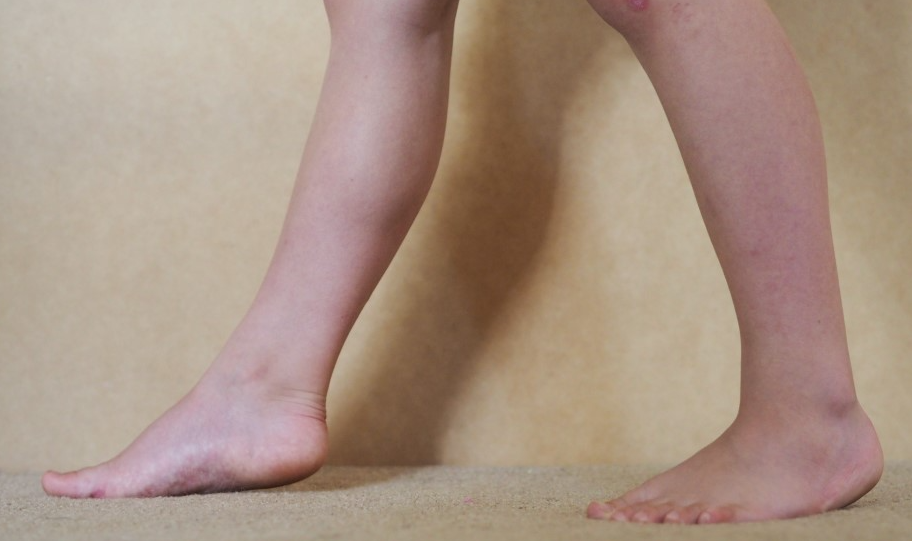 | 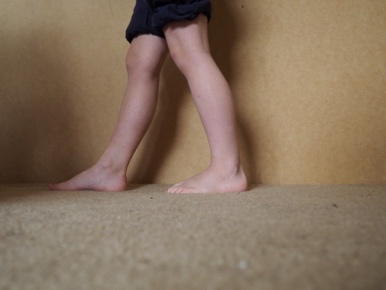 | 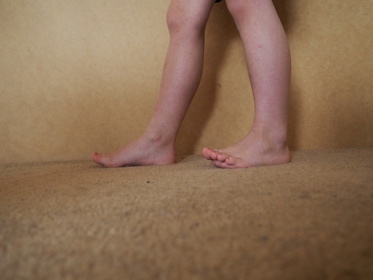 | 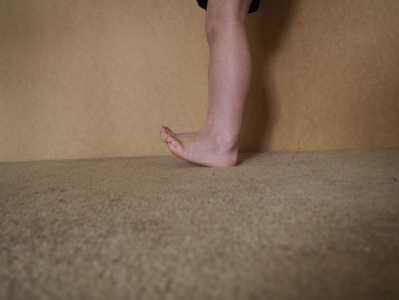 |
| --- | --- | --- | --- | --- |
| 0 – Topuğu veya orta ayağı yere değdiremiyor. | 1 – Topuğunu yere getiremiyor. Orta ayak vuruşu mevcut. | 2. Topuğu ile basabiliyor. Ayak baş parmak dorsifleksiyonda  10 adım atamıyor. | 3. Topukla basabiliyor ve ayak baş parmak dorsifleksiyonda 10 adım atabiliyor. Fakat ön ayak tabanı yere temas etmeye devam ediyor. | 4. Topuğu ile basabiliyor ve ön ayak tabanını yerden kaldırarak 10 adım atabiliyor. |

***Yürüme Hızı (uygun olan şekilde)***

**6 DYT:**

**Süre: 2 dk (dk/sn):______ Süre: 4 dk (dk/sn):_____ Süre: 6 dk (dk/sn):_____**

**Toplam süre (dk/sn):_______**
