# Supplementary material for: Validity and Reliability of the Turkish Version of the Idiopathic Toe Walking Outcome (iTWO) Proforma
Source: J Foot Ankle Res. 2026 Jul 28;19(3):e70188. doi: 10.1002/jfa2.70188 (PMC13415977; doi:10.1002/jfa2.70188)
Supplement: Supplementary file 2 — Table S1: Correlation analyses demonstrating the validity of the iTWO proforma‐T. [file JFA2-19-e70188-s002.docx]

**Table S1.** Correlation analyses demonstrating the validity of the iTWO proforma-T.

|  |  | PODCI-UE | PODCI-TBM | PODCI-SPF | PODCI-PC | PODCI-H | PODCI-GF | OxAFQ-PF Child | OxAFQ-SP Child | OxAFQ-EWB Child | OxAFQ-FW Child | OxAFQ-PF Parent | OxAFQ-SP Parent | OxAFQ-EWB Parent | OxAFQ-FW Parent |
| --- | --- | --- | --- | --- | --- | --- | --- | --- | --- | --- | --- | --- | --- | --- | --- |
| Question 1 | rho | .074 | .002 | -.050 | .199 | -.004 | .121 | -.050 | -.242 | -.493^**^ | -.069 | -.233 | -.103 | -.409^*^ | -.157 |
|  | p | .671 | .990 | .777 | .252 | .983 | .489 | .776 | .161 | .003 | .693 | .178 | .557 | .015 | .367 |
| Question 2 | rho | .044 | -0.099 | .141 | .168 | .094 | .135 | -.085 | .142 | .431^**^ | .099 | .295 | .213 | .243 | .112 |
|  | p | .801 | .572 | .419 | .334 | .592 | .438 | .629 | .417 | .010 | .571 | .085 | .219 | .160 | .523 |
| Question 3 | rho | -.286 | -.032 | -.073 | -.109 | -.018 | -.169 | .130 | .100 | .031 | -.169 | .004 | .201 | .051 | -.093 |
|  | p | .096 | .854 | .675 | .533 | .920 | .332 | .456 | .568 | .857 | .331 | .981 | .247 | .771 | .596 |
| Question 4 – Today | rho | -.126 | -.294 | -.470^**^ | -.161 | -.183 | -.329 | -.263 | .047 | -.213 | -.151 | -.381^*^ | .018 | -.231 | -.144 |
|  | p | .472 | .086 | .004 | .356 | .291 | .054 | .127 | .788 | .220 | .388 | .024 | .918 | .182 | .410 |
| Question 4 – Activity | rho | -.113 | -.514^**^ | -.249 | -.113 | -.050 | -.153 | -.232 | -.220 | -.243 | -.134 | -.296 | -.247 | -.305 | -.228 |
|  | p | .518 | .002 | .148 | .518 | .776 | .381 | .179 | .204 | .159 | .444 | .084 | .153 | .075 | .187 |
| WBL, knee straight, Right | rho | .056 | -.039 | .078 | -.084 | -.077 | .004 | -.049 | -.123 | .191 | .170 | .012 | .002 | .082 | .262 |
|  | p | .750 | .823 | .656 | .632 | .662 | .980 | .779 | .481 | .271 | .329 | .946 | .993 | .640 | .128 |
| WBL, knee bent, Right | rho | -.038 | -.097 | -.041 | -.215 | .013 | -.161 | .058 | -.025 | .318 | .264 | .047 | -.079 | .227 | .231 |
|  | p | .827 | .580 | .814 | .215 | .943 | .354 | .741 | .885 | .063 | .126 | .787 | .654 | .189 | .183 |
| Gait Scale | rho | -.256 | .046 | .117 | -.180 | -.035 | -.097 | .064 | -.078 | -.030 | -.111 | .003 | -.136 | -.008 | -.096 |
|  | p | .138 | .795 | .505 | .300 | .844 | .581 | .716 | .657 | .863 | .527 | .988 | .435 | .961 | .582 |
| 2 minute | rho | .162 | .069 | -.063 | -.103 | -.086 | -.065 | -.098 | -.097 | .337 | -.065 | .078 | .068 | .196 | .101 |
|  | p | .360 | .699 | .722 | .563 | .630 | .714 | .582 | .587 | .051 | .715 | .659 | .704 | .266 | .568 |
| 4 minute | rho | .151 | .344^*^ | -.052 | -.103 | -.057 | -.021 | -.019 | -.049 | .169 | .090 | .046 | .051 | .114 | .073 |
|  | p | .958 | .947 | .910 | .395 | .046 | .768 | .561 | .749 | .908 | .917 | .782 | .341 | .523 | .683 |
| 6 minute | rho | .156 | .161 | .095 | -.006 | .007 | .101 | -.118 | -.170 | .057 | -.037 | -.007 | .157 | .057 | .168 |
|  | p | .379 | .364 | .593 | .975 | .970 | .571 | .505 | .338 | .749 | .834 | .969 | .375 | .747 | .342 |
| TWSS | rho | .047 | .106 | .109 | -.131 | -.022 | -.030 | .051 | .127 | .117 | -.001 | .074 | .170 | .014 | .110 |
|  | p | .789 | .543 | .534 | .454 | .901 | .866 | .769 | .466 | .503 | .997 | .671 | .330 | .934 | .528 |
| *p < 0.05; **p < 0.01. OxAFQ-PF: The Oxford Ankle Foot Questionnaire – physical function; OxAFQ-SP: school and play; OxAFQ-EWB: emotional well-being; OxAFQ-FW: footwear; PODCI-SPF: Pediatric Outcomes Data Collection Instrument – sports and physical function; PODCI-PC: pain/comfort; PODCI-UE: upper extremity function; PODCI-TBM: transfer and basic mobility; PODCI-H: happiness; PODCI-GF: global functioning; WBL: weight-bearing lunge. | | | | | | | | | | | | | | | |
